# Unbalanced Expression of ICOS and PD-1 in Patients with Neuromyelitis Optica Spectrum Disorder

**DOI:** 10.1038/s41598-019-50479-4

**Published:** 2019-10-01

**Authors:** Qun Xue, Xiaoping Li, Yanzheng Gu, Xiaozhu Wang, Mingyuan Wang, Jingluan Tian, Xiaoyu Duan, Hanqing Gao, Xiaopei Ji, Xiaoming Yan, Wanli Dong, Qi Fang, Xueguang Zhang

**Affiliations:** 1 Department of Neurology, First Affiliated Hospital of Soochow University, Suzhou, Jiangsu 215006 China; 2 Institute of Clinical Immunology, Jiangsu Key Laboratory of Clinical Immunology, First Affiliated Hospital of Soochow University, Suzhou, Jiangsu 215006 China; 3 Suzhou Clinical Medical Center of Neurology, Suzhou, Jiangsu 215004 China; 4 Suzhou Red Cross Central Blood Station, Suzhou, Jiangsu 215006 China

**Keywords:** Neuroimmunology, Demyelinating diseases

## Abstract

Neuromyelitis optica spectrum disorder (NMOSD) likely results from humoral immune abnormalities. The role that helper T cells play in the pathogenesis of this disease is not fully understood. To ascertain the clinical significance of two important costimulatory molecules required for T-cell activation in the peripheral blood of patients with NMOSD, we examined the expression levels of a membrane- and soluble-type inducible costimulatory molecule (ICOS), its ligand (ICOSL), programmed death-1 (PD-1), and its ligand (PD-L1) in the peripheral blood of 30 patients with NMOSD and compared these levels with those in patients with longitudinally extensive transverse myelitis (LETM), those with optic neuritis (ON), and healthy controls (HCs). Our results showed that the ICOS/ICOSL and PD-1/PD-L1 pathways may play important roles in the early stages of NMOSD pathogenesis. ICOS and PD-1 are potential therapeutic targets and valuable biomarkers for the differential diagnosis of early-stage NMOSD.

## Introduction

Neuromyelitis optica spectrum disorder (NMOSD) refers to a class of demyelinating diseases of the central nervous system (CNS) characterized by longitudinally extensive transverse myelitis (LETM) and severe optic neuritis (ON). It generally occurs among young women and is associated with a high disability rate. Because of its association with the specific antibody aquaporin 4-IgG (AQP4-IgG), NMOSD was previously thought to be a CNS autoimmune disease primarily mediated by humoral immunity^1,2^. However, B cell depletion therapy does not alleviate the disease in all cases. In addition, the NMOSD pathological process cannot be completely explained by humoral immune abnormalities in antibody-negative patients with NMOSD. Recently, in-depth research concerning NMOSD pathogenesis and disease models have shown it to be a neurological immune disease with the involvement of both T lymphocytes and B lymphocytes. Therefore, T lymphocyte immunity might play an important role in the early stages of this disease^3,4^.

T cell activation requires the major histocompatibility complex (MHC)-peptide complex to provide an initial signal as well as a second signal delivered by costimulatory molecules. A lack of costimulatory signals can result in T cells that are unable to respond, as well as programmed cell death. The molecules that mediate costimulatory signals are called “costimulatory molecules”, and they are primarily divided into two superfamilies: the CD28/B7 superfamily and the TNFR/TNF (tumor necrosis factor receptor/tumor necrosis factor) superfamily^5–7^. These superfamilies can be divided into positive and negative costimulatory molecules based on their immunomodulatory effects. The inducible costimulatory molecule (ICOS) and its ligand (ICOSL) are important members of the CD28/B7 family. Recently, they have received extensive attention in peripheral immune tolerance and autoimmune injury research. However, the research concerning the role and mechanism of this pathway in the pathological process of NMOSD remains in its infancy. ICOS is induced in activated T cells. It promotes the formation of germinal centers and effective interactions between T and B cells. Immunoglobulin isotype switching is attenuated in ICOS-deficient mice, and ICOS/ICOSL primarily transmits positive signals^8^. By contrast, the other pair of molecules, programmed death-1 (PD-1) and its ligand (PD-L1), are a pair of costimulatory molecules recently identified as primarily involved in negative immune modulation. They play crucial roles in the progression of many diseases. PD-L1, also known as B7-H1, produces an inhibitory signal after binding to its receptor, inducing T cell apoptosis and inhibiting T cell activation and proliferation^5,9^, thereby negatively regulating the immune response and participating in the regulation of immune tolerance, responses to microbial infection, and tumor immune evasion^10,11^. In addition to their roles in immuno-oncology mechanisms, which have received broad attention, the roles of these costimulatory molecules in autoimmune diseases and the application of related monoclonal antibodies in the treatment of these diseases have increasingly become a hot research topic. Among these studies, the research on PD-1/PD-L1 in autoimmune diseases such as rheumatoid arthritis and psoriasis has achieved significant results^12–14^.

Even with the latest NMOSD diagnostic criteria, it remains difficult to differentiate aquaporin 4-IgG (AQP4-IgG)-negative patients with early-stage NMOSD from those with ON, LETM, or multiple sclerosis (MS). ICOS/ICOSL and PD-1/PDL-1 are important molecules that regulate T lymphocytes, and they might be associated with pathological changes, such as immune microenvironment disorder, the dangerous upregulation of humoral immunity, and cellular immunity, in patients with NMOSD. Therefore, exploring their possible roles and mechanisms in NMOSD immunopathology is important for elucidating the pathological mechanism of this disease, its qualitative and timely diagnosis, and searching for new targets for therapeutic intervention.

The current study employed immunofluorescence labeling and flow cytometry to examine the expression levels of membrane type ICOS (mICOS), membrane type ICOSL (mICOSL), membrane type PD-1 (mPD-1), and membrane type PD-L1 (mPD-L1) on the surface of peripheral T cells, B cells, and monocytes cells in patients with NMOSD (NMOSD group) and compared these levels with those in patients with LETM (within 1 week of disease onset; the LETM group), patients with ON (within 1 week of disease onset; the ON group), and healthy controls (the HC group). Additionally, the levels of sICOS, sICOSL, sPD-1, and sPD-L1 in serum samples were examined using enzyme-linked immunosorbent assays (ELISAs) for each group. This study analyzed the immunopathological effects of the ICOS/ICOSL and PD-1/PD-L1 pathways during the early stages of NMOSD and assessed the potential utility of these molecules as biomarkers for the auxiliary diagnosis of NMOSD.

## Methods

### Ethical statement

Serum and peripheral blood samples were obtained from the participants after they provided informed consent based on the protocol approved by the local ethics committees of Soochow University and its First Affiliated Hospital (ethics approval number 2013-055). Informed consent was waived for the control volunteers.

### Sample collection

Patients were recruited from outpatient and inpatient populations treated at the Department of Neurology of the First Affiliated Hospital of Soochow University between April 2015 and December 2017, including 30 patients with NMOSD, 30 patients with LETM, and 16 patients with ON. Sixteen HC participants were recruited from the physical examination center of the First Affiliated Hospital of Suzhou University. All controls were healthy adults without histories of autoimmune diseases, infection, or allergies who had not received hormone or immunosuppressive agent treatments within 3 months prior to the study. According to the diagnostic and classification criteria revised by the International Panel for Neuromyelitis Optica Diagnosis (IPND) in 2015, patient diagnosis was confirmed based on typical clinical manifestations, serum AQP4-IgG test results, and imaging findings. Based on its definition, LETM refers to acute myelitis involving damage in three or more consecutive spinal segments. Patients with LETM who had negative serum AQP4-IgG test results and did not meet the NMOSD criteria were enrolled. Patients with ON were enrolled based on the diagnostic criteria proposed in the 2014 Consensus Guidelines for the Diagnosis and Treatment of ON. The enrolled patients with NMOSD, LETM, or ON were all newly diagnosed and had not been previously treated with immunosuppressive agents. Of the 30 patients diagnosed with NMOSD, 28 tested positive for AQP4-IgG, and two tested negative for AQP4-IgG. AQP4-IgG testing was performed for all patients and HCs using an ELISA kit (RSR, UK) and a cell-based assay (CBA) method performed by our group or Euroimmun CN, Inc.

A 4-mL volume of venous blood was collected from the HC, NMOSD, LETM, and ON groups using tubes containing ethylenediaminetetraacetic acid (EDTA) as an anticoagulant, followed by centrifugation at 1,000 × g for 20 minutes. The plasma in the upper layer was collected and stored at −80 °C until subsequent use.

### Immunofluorescence labeling and flow cytometry

Based on the user’s manuals for the reagents, i.e., the FITC-labeled mouse anti-human CD4 antibody (Beckman Coulter, CA), FITC-labeled mouse anti-human CD14 antibody (Biolegend, CA), FITC-labeled mouse anti-human CD19 antibody (Biolegend, CA), PE-labeled mouse anti-human ICOS antibody (eBioscience, CA), PE-labeled mouse anti-human ICOS-L antibody (Biolegend, CA), PerCP/Cy5.5 anti-human CD279 (PD-1; Biolegend, CA), PE-labeled mouse anti-human PD-L1 antibody (Biolegend, CA), and PE/Cy7 anti-human CD185 (CXCR5) antibody (Biolegend, CA), each reagent was separately added to 50 μL of whole blood, followed by incubation in the dark for 30 minutes at room temperature. To each tube, we added 200 μL of red blood cell lysis buffer (Beckman Coulter, CA), mixed the solutions thoroughly through pipetting, and incubated them in a 37 °C water bath for 10 minutes for complete lysis followed by the addition of 1 mL of phosphate-buffered saline (PBS) to stop the lysis. The lysate was then centrifuged at 1,800 rpm for 5 minutes at room temperature, and the supernatant was discarded. Each tube was resuspended with 400 μL of PBS, mixed thoroughly through pipetting, and subjected to testing using a flow cytometer (Beckman, CA). These data were analyzed using FlowJo version 7.6.

### ELISAs

Serum samples were removed from the −80 °C freezer, thawed at room temperature, centrifuged at 15,000 × g for 5 minutes, and then allowed to stand until further use. The specific experimental procedure followed the manufacturer’s protocols for the quantitative ELISA kits regarding sICOS (Shanghai Yu Bo Biotech, China), sICOSL (Suzhou Bright Scistar Biotechnology, China), sPD-1, sPD-L1 and Interleukin 21 (IL-21; all from Shanghai Kang Lang Biotechnology), and AQP4-IgG (RSR Limited, UK). We added 50 μL of patient serum and 50 μL of biotinylated antibody-linked active enzyme to the corresponding wells (excluding the blank wells). The plate was then covered with a sealing membrane and incubated for 1–2 hours at room temperature. The liquid in the wells was discarded, and the plate was washed three times and dried. To each well, we added 100 μL of 3,3′, 5,5′-tetramethylbenzidine (TMB) substrate solution and incubated the plates at room temperature for 30 minutes in the dark, without shaking, followed by the addition of 100 μL of stop solution to each well. The absorbance of each well at 450 nm was measured using a microplate reader (Multiskan MK3, Thermo, Germany). The concentration of AQP4-IgG was measured, and the AQP4-IgG results were validated through comparison with a cell-based analysis (CBA) and the indirect immunofluorescence (IIF) assay of Oumeng Diagnostics.

### Statistical analyses

Statistical analyses were performed using SPSS version 19.0. Normally distributed measurement data were expressed as means ± standard deviations (x ± s), and nonnormally distributed measurement data were expressed as medians (interquartile range). The Wilcoxon rank sum test was used to compare the means between two samples with normally distributed data, and the Kruskal-Wallis H test was used to compare the means among multiple samples with nonnormally distributed data. Intergroup comparison was performed using the Wilcoxon rank sum test after a Bonferroni correction. Correlation tests were performed using Pearson’s correlation analysis. P < 0.05 was considered as significant.

## Results

Table 1 shows detailed information regarding the sample. Table 1:

**Table 1:** Detailed information regarding the patient population.

	HC	NMOSD	LETM	ON
	n = 16	n = 30	n = 30	n = 16
Age	37.95 ± 2.15	37.60 ± 3.83	46.78 ± 5.09	30.62 ± 5.72
Gender (Female/male)	8/8	18/12	16/14	8/8
ELISA-AQP4-IgG (U/L)	0.68 ± 0.12	23.09 ± 8.94	2.04 ± 1.73	1.73 ± 0.93
CBA-AQP4-IgG	NA	1:32–1:1024	1:4–1:8	1:4–1:16
Positive/negative for AQP-4 IgG	0/16	28/2	0/30	0/16
EDSS	0	3.93 ± 2.18	5.02 ± 2.37	2.02 ± 0.50

### The upregulation of the expression of ICOS/ICOS-L and PD-1/PDL-1 in the immune cells of patients with NMOSD

**Figure 1:**
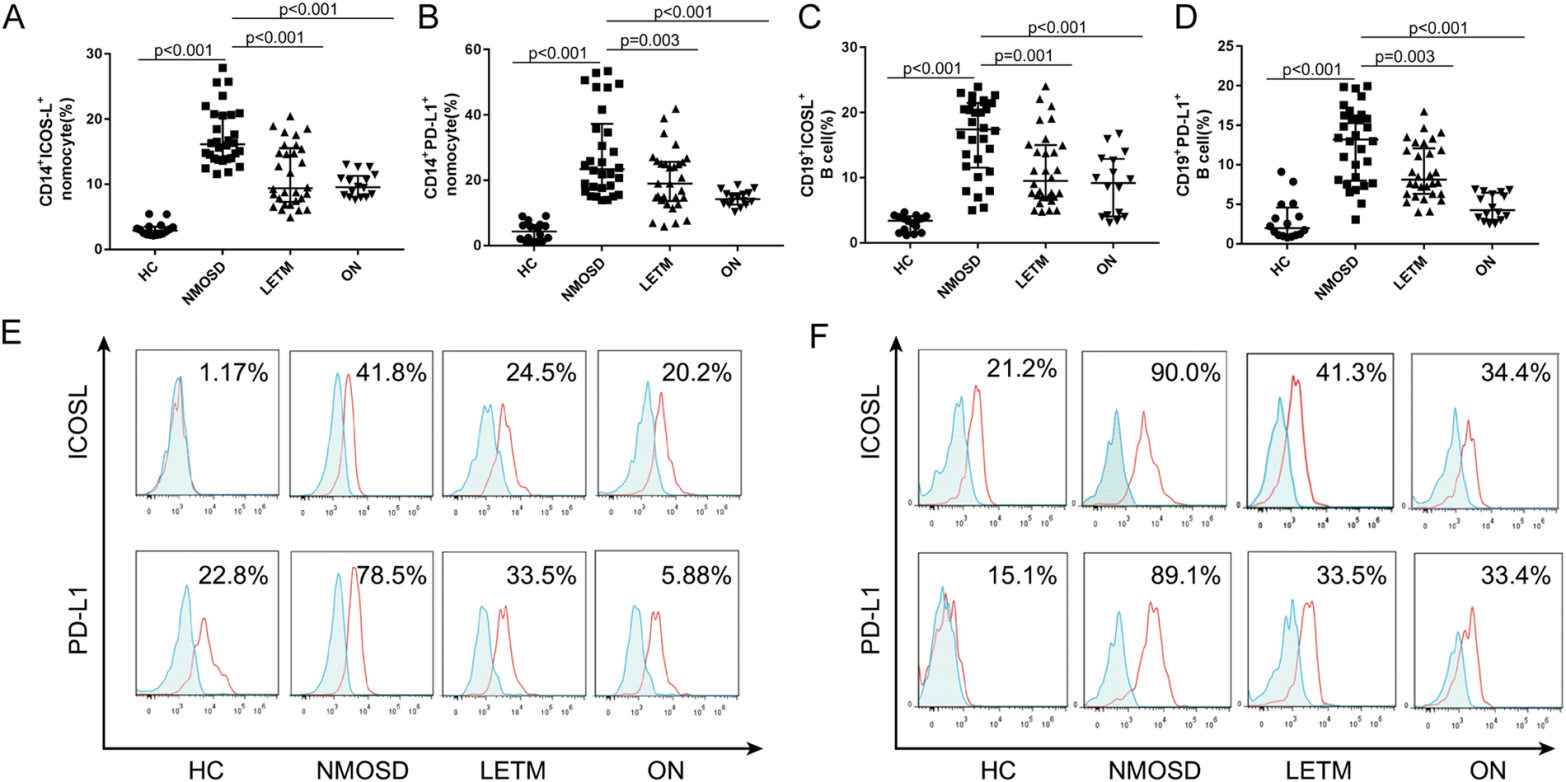
The expression levels of ICOS and PD-1 on the surface of the peripheral CD4+ T cells in the peripheral blood of patients with NMOSD. The expression levels of ICOS and PD-1 in the peripheral CD4+ T cells of patients with and without NMOSD were detected via immunofluorescence labeling and flow cytometry. On the graphs (**A**,**B**) and histogram (**C**), the gray areas represent the negative control, and the red lines indicate the test results for the costimulatory molecules ICOS and PD-1. Note: HC, healthy control group; NMOSD, neuromyelitis optica spectrum disorder group; LETM, longitudinally extensive transverse myelitis group; ON, optic neuritis group; ICOS, inducible costimulatory molecule; PD-1, programmed death-1.

The membrane-type costimulatory molecules on the peripheral mononuclear cells were examined in the four study groups (HC, NMOSD, LETM, and ON) using immunofluorescence labeling and flow cytometry analyses. The results showed that the expression levels of ICOS and PD-1 in the peripheral CD4+ T lymphocytes of patients with NMOSD were significantly higher than those of the other three groups (P < 0.05; the specific values are shown in Fig. 1).

**Figure 2:**
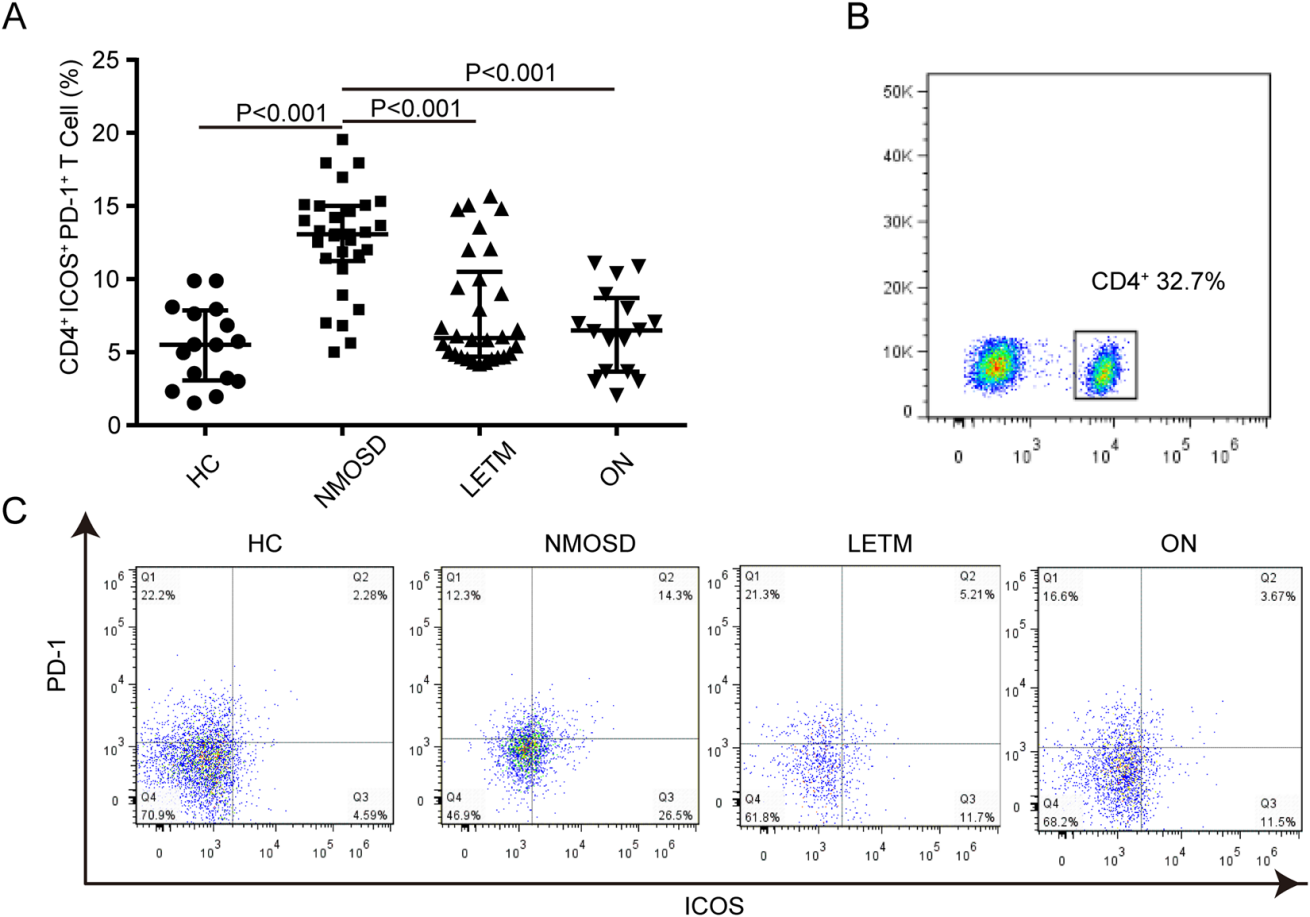
Expression levels of ICOSL and PD-L1 on the surfaces of CD14+ monocytes and CD19+ B cells in the peripheral blood of patients with NMOSD. The ICOSL expression on CD14+ monocytes (graph **A**), the PD-L1 expression on CD14+ monocytes (graph **B**), the ICOSL expression on CD19+ B cells (graph **C**), the PD-L1 expression on CD19+ B cells (graph D), and the expression levels of ICOSL and PD-L1 on CD14+ monocytes (histogram E) and CD19+ B cells (histogram F) in the peripheral blood of patients with and without NMOSD were detected through immunofluorescence labeling and flow cytometry. Gray areas represent the negative control, and the red lines indicate the test results of the costimulatory molecules ICOSL and PD-L1. Note: HC, healthy control group; NMOSD, neuromyelitis optica spectrum disorder group; LETM, longitudinally extensive transverse myelitis group; ON, optic neuritis group; ICOSL, inducible costimulatory ligand; PD-L1, programmed death 1-ligand.

The expression levels of ICOSL and PD-L1 on the surfaces of the peripheral CD14+ monocytes and CD19+ B lymphocytes in patients with NMOSD were significantly higher than those in the other three groups (P < 0.05; the specific values are shown in Fig. 2).

**Figure 3:**
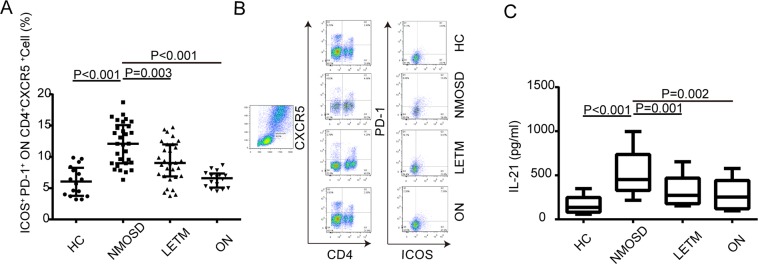
The coexpression levels of ICOS and PD-1 on the surface of CD4+ T cells in the peripheral blood of patients with NMOSD. The expression levels of ICOS and PD-1 (graph **A** and scatterplots **B** and **C**) in the peripheral blood CD4+ T cells of patients with and without NMOSD were detected through immunofluorescence labeling and flow cytometry. The area within the frame represents the CD4+ T cells. Note: HC, healthy control group; NMOSD, neuromyelitis optica spectrum disorder group; LETM, longitudinally extensive transverse myelitis group; ON, optic neuritis group; ICOS, inducible costimulatory molecule; PD-1, programmed death-1.

The ICOS and PD-1 coexpression levels of the CD4^+^ T lymphocytes in the peripheral blood samples of patients with NMOSD were significantly higher than those in the other three groups (P < 0.05; the specific values are shown in Fig. 3).

### The upregulation of the expression of follicular B helper T cells (Tfh cells) and the expression level of IL-21 in patients with NMOSD

**Figure 4:**
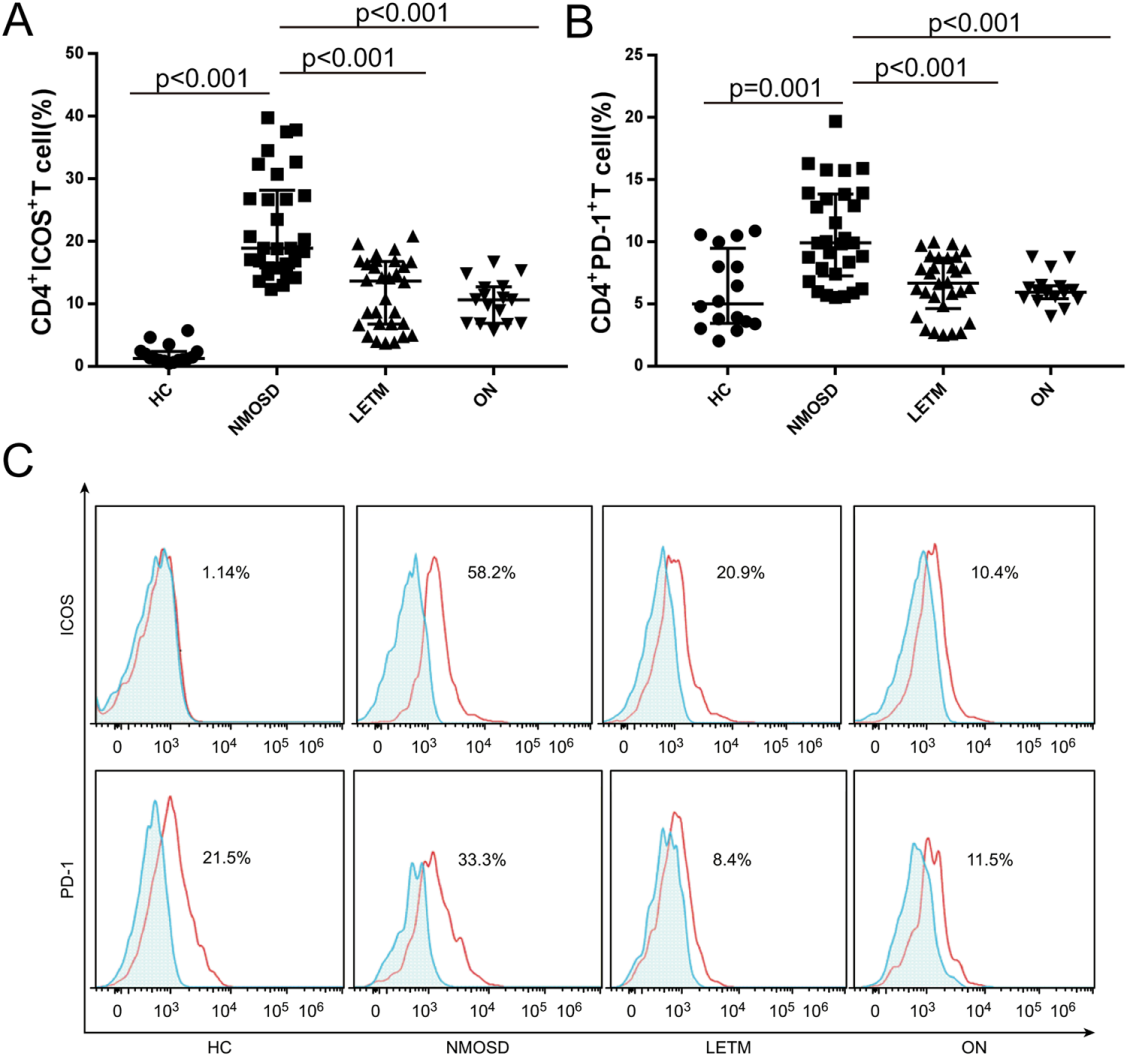
The expression levels of Tfh cells in the peripheral blood of patients with NMOSD. The expression levels of CXCR5, ICOS, and PD-1 (graph **A** and scatterplots **A**,**B**) in the peripheral blood CD4+ T cells of patients with and without NMOSD were detected through immunofluorescence labeling and flow cytometry. The area within the frame represents CD4+ T cells. (**C**) IL-21 expression levels of the patients from each group. Note: HC, healthy control group; NMOSD, neuromyelitis optica spectrum disorder group; LETM, longitudinally extensive transverse myelitis group; ON, optic neuritis group; CXCR5, C-X-C chemokine receptor type 5; ICOS, inducible costimulatory molecule; PD-1, programmed death-1; IL-21, interleukin-21.

The coexpression levels of mCXCR5, mICOS, and mPD-1 on CD4+ T lymphocytes (Tfh cells) as well as the expression of IL-21 in the peripheral blood samples of patients with NMOSD were significantly higher than those in the other three groups (P < 0.05; the specific values are shown in Fig. 4).

### The upregulation of sICOSL, sPD-1, and sPD-L1 and the downregulation of sICOS expression in patients with NMOSD

In addition to being expressed as membrane-bound molecules on the surface of the cell membrane, ICOS and ICOSL exist in soluble forms. The expression levels of sICOS and sICOSL in the peripheral blood of each group were examined using ELISA. The results showed significantly increased sICOSL expression and decreased sICOS expression in the serum of patients with NMOSD compared with the other three groups (P < 0.05, Table 1).

PD-1 and PD-L1 also have soluble forms. We further examined the expression levels of sPD-1 and sPD-L1 in the peripheral blood of each group. The results showed significantly elevated expression levels of sPD-1 and sPD-L1 in the peripheral blood of patients with NMOSD compared with the other three groups (P < 0.05, Table 2).

**Table 2:** Expression levels of the soluble costimulatory molecules sICOS, sICOSL, sPD-1, and sPD-L1 in the serum of patients with NMOSD (mean ± standard deviation).

	*Number*	sICOS *(pg/mL)*	sICOSL *(pg/mL)*	sPD-1 *(pg/mL)*	sPDL-1 *(pg/mL)*
*HC*	16	18.33 (14.71)	6.26 (3.82)	34.06 (10.56)	40.20 (7.20)
*NMOSD*	30	8.80 (9.57)^abc^	14.97 (9.29)^abc^	58.69 (12.77)^abc^	50.05 (26.67)^abc^
*LETM*	30	17.09 (6.63)	9.01 (6.27)	39.58 (14.51)	36.31 (9.19)
*ON*	16	16.09 (6.00)	5.48 (4.70)	38.47 (11.53)	39.31 (2.98)
*a*	*NMOSD group vs. HC group*	*t* = *3.221, p* = *0.001*	*t* = *2.932, p* = *0.003*	*t* = *3.751, p* < *0.001*	*t* = *2.986, p* = *0.003*
*b*	*NMOSD group vs. LETM group*	*t* = *2.399, p* = *0.016*	*t* = *2.698 p* = *0.007*	*t* = *2.665 p* = *0.008*	*t* = *3.385, p* = *0.001*
*c*	*NMOSD group vs. ON group*	*t* = *2.557, p* = *0.009*	*t* = *2.843, p* = *0.003*	*t* = *2.932, p* = *0.003*	*t* = *3.567, p* < *0.001*

Note: sICOS, soluble ICOS; sICOSL, soluble ICOSL; sPD-1, soluble PD-1; sPD-L1, soluble PD-L1.

The sICOS/sICOSL ratio of the NMOSD group was lower than those in the LETM, ON, and HC groups (P < 0.05), but no significant differences were observed regarding the sPD-1/sPD-L1 ratio between the NMOSD group and the other three groups (P > 0.05).

## Discussion

With the discovery of AQP-4 IgG, the consensus regarding the pathogenesis of NMOSD has shifted from a strictly autoimmune condition to a CNS demyelination disease mediated primarily by humoral immunity. NMOSD is distinct from MS given the differences in their pathogeneses, clinical manifestations, imaging results, and prognoses. In-depth research on the pathogenic mechanisms has determined that AQP-4 IgG cannot fully explain the pathological process of NMOSD, primarily with regard to the following aspects: (1) AQP4-IgG infused into the peripheral blood of mice does not completely pass the blood-brain barrier and cannot cause disease in mice; however, following the destruction of the blood-brain barrier in experimental mice using the pertussis virus and lipopolysaccharide, infused AQP-4 IgG did not reproduce the disease condition. Therefore, AQP-4 IgG by itself likely does not have the ability to destroy the blood-brain barrier, and an infusion of AQP-4 IgG does not induce an immune response in the CNS. Under nonspecific conditions, AQP-4 IgG cannot fully exert its pathogenic effects in the CNS^15–17^. Hence, other inflammatory response mechanisms might be present in the pathogenesis of NMOSD, including those that destroy the blood-brain barrier and promote the pathogenic effects of AQP-4 IgG. (2) Clinically, some patients test negative for AQP-4 IgG, even though they have clinical symptoms of NMOSD. Wingerchuk *et al*.^3^ specifically listed the AQP4-IgG negative subtype in their published consensus guidelines for the NMOSD diagnostic criteria in 2015. (3) B cell depletion therapy does not clinically alleviate the disease conditions in all patients, suggesting the presence of other immunological molecular mechanisms to be elucidated and humoral immune mechanisms such as the pathogenic antibody AQP4-IgG.

Through the collection of peripheral T cells from patients with NMOSD and healthy adults as well as via blocking of the surface allele of the AQP4 antigen, Varrin-Doyer *et al*.^18^ demonstrated that the T cells of patients with NMOSD differentiate into specific T cells under the coordinated actions of costimulatory molecules (e.g., CD80 and CD40) and cytokines (e.g., IL-6 and IL-17), thereby producing an inflammatory response in the CNS that causes the onset of NMOSD. This finding supports the theory that cellular immunity plays an essential role in the pathogenesis and progression of NMOSD. Based on this study, Zeka *et al*.^19^ used the same method to block the AQP4 antigen and further confirmed that specific T cells against different epitopes were reactivated after crossing the blood-brain barrier and exerted their pathogenic effects through the infiltration of the optic nerve, brain, and spinal cord. Specific T cells, together with NMO-IgG, can damage the large astrocytes located primarily in the gray matter of the spinal cord^19^. This specific activation of T cells serves as a necessary prerequisite for AQP-4 IgG to enter the CNS and exert its pathogenic effect during the early stages of NMOSD, and the related costimulatory molecules might play certain immunopathological roles in the pathological process of NMOSD^20–23^.

Recent research on costimulatory molecules has made rapid progress. The B7-CD28 family is the most well-studied family of costimulatory signaling molecules that have been recognized. Numerous studies have shown that aberrantly expressed costimulatory molecules participate in the pathological processes of multiple autoimmune diseases, including rheumatoid arthritis, systemic lupus erythematosus, asthma, and kidney disease in different ways by mediating the abnormal activation of immune cells during different phases of the immunopathological response^24–28^. Costimulatory molecules also play certain roles in organ transplantation, anti-infection response, and immuno-oncology^29^. According to their different immunomodulatory activities, this class of molecules can be divided into positive and negative costimulatory molecules, and their regulatory networks play an extremely important regulatory role in the effective initiation, modulatory effects, and timely termination of immune responses^30^. Signaling abnormalities related to these molecules often disrupt immune homeostasis in the body, resulting in disease development.

The ICOS/ICOSL signaling pathway provides a positive immune signal. Its pathologically enhanced expression leads to the disruption of immune regulation and self-tolerance. Its signaling can induce the secretion of cytokines related to Th1 and Th2, including IFN-γ, TNF-α, IL-4, IL-5, and IL-10. The ICOS/ICOSL pathway has a dominant effect on Th2 immune responses; it not only promotes the expression of cytokines related to Th2 polarization but also acts on the Th2-associated transcription factors NFATcl and C-Maf^5,6,8,31,32^. The current study demonstrated that patients with NMOSD had significantly higher expression levels of membrane-type ICOS/ICOSL and sICOSL in their peripheral blood than those in the LETM, ON, or HC groups; moreover, patients with NMOSD had a significantly lower concentration of sICOS than the other three groups. The sICOS/sICOSL ratio of the NMOSD group was significantly lower than those of the other three groups. The mean sICOSL level in the peripheral blood of patients with NMOSD was relatively overabundant. sICOSL is a functional molecule^33^ that might provide more positive stimulatory signals to mICOS in T cells, overactivate T cells, and participate in the pathological process of NMOSD.

PD-1/PD-L1, which also belongs to the B7 family, provides a negative immunomodulatory signal. The binding of the PD-1 on T cells to the PD-L1 on antigen-presenting cells inhibits T-cell receptor (TCR)-mediated lymphocyte activation, differentiation, and proliferation as well as the production of cytokines (e.g., IL-2, IFN-γ, and IL-10), thereby causing cell cycle arrest in T cells. PD-1/PD-L1 interactions play important regulatory roles during the initial phases of the activation and expansion of autoreactive T cells and in the secondary immune responses of T cells; moreover, they inhibit B cell proliferation, differentiation, and Ig type conversion. Because of the broad expression profile of its ligand, PD-L1, PD-1 is likely a key regulatory component of lymphocyte activation in autoimmune diseases^9,10^. PD-1/PD-L1 interfere with the normal function of cellular immunity and contribute to tumor evasion^34,35^. Autoimmune disease studies have shown the upregulated expression of both membrane-type and soluble forms of PD-1/PD-L1 during the early stages of the disease, but their expression levels return to normal during later and more stable stages of the disease^36,37^. The current study showed significantly elevated expression levels of mPD-1 in CD4+ T cells and the ligand PD-L1 on the surfaces of the CD19+ B lymphocytes and CD14+ monocytes in the peripheral blood of patients with NMOSD during the early stages of the disease compared with the other three groups. These findings are similar to those previously reported regarding the upregulation in patients with rheumatoid arthritis. This similarity indicates that the disease was in an active phase, and the altered expression levels were correlated with the severity of the disease^38^. This correlation raises an important question: Why does PD-1/PD-L1 not function properly and transmit negative signals? We examined the soluble forms of PD-1 and PD-L1. Other studies have demonstrated that sPD-1 and sPD-L1 inhibit the PD-1/PD-L1 pathway and enhance the activity of T cells, which might benefit the early treatment of multiple diseases such as autoimmune hepatitis and childhood autoimmune arthritis^12,39,40^. In this study, the sPD-1 and sPD-L1 levels in the peripheral blood of patients with early-stage NMOSD were significantly higher than those of the other three groups. (1) This difference might somewhat interfere with the negative signal transduction of mPD-1/mPD-L1, destroying immunomodulation homeostasis^41^. The degree of glycosylation of these molecules remains unclear and deserves additional study. (2) The source of sPD-1/sPD-L1 in patients with NMOSD is not well understood. The specific elevations of sPD-1 and sPD-L1 are likely correlated with the increases in mPD-1 and mPD-L1. The specific increase in these soluble molecules might be a compensatory mechanism in response to the overexpression of the membrane-type molecules. (3) sPD-L1 and sPD-1 were expressed at a similar ratio in the serum of patients after they increased, and no relative excess was found of either molecule in the blood. Although their concentrations were significantly higher than those of the other groups, the ligand-to-receptor ratio remained similar. Therefore, their increases had relatively small interference effects on membrane-type PD-1 or PD-L1, which contrasts with the apparent excess of sICOSL relative to sICOS, in which sICOSL holds a relatively dominant status and might have more opportunities to bind to the ICOS on T cell membranes. This imbalance between positive and negative signal expression might be an important mechanism in the pathological process of NMOSD. The dynamic monitoring of the changes in the peripheral expression levels of the costimulatory molecules in patients and *in vivo* studies using animal models might help determine whether the imbalanced expression of positive and negative costimulatory molecules can be corrected by interfering with the ICOS/ICOSL and PD-1/PD-L1 signals.

In this study, the co-expression of the positive costimulatory molecule ICOS and the negative costimulatory molecule PD-1 was examined on the CD4+ T cell membrane for the first time in patients with a CNS immune disease. The results showed that the ratio of CD4+ ICOS+ PD-1+ T cells in the peripheral blood of patients with newly diagnosed early-stage NMOSD was significantly higher those in three other study groups. Considering the above test results for the soluble molecules and IL-21; the available literature regarding the secretion and co-expression of CXCR5, ICOS, and PD-1 by Tfh cells; and the expression of Tfh in patients with NMOSD^4^, we speculate that the abnormal increase of this subpopulation in the peripheral blood of patients with NMOSD probably transmits a positive signal to B cells, causing abnormal B cell activation and secretion of numerous pathogenic antibodies. This subpopulation of cells might have potential diagnostic value for the early differentiation of NMOSD from LETM and ON; however, it is necessary to further enlarge the sample size, determine the range of definitive values, and conduct long-term follow-up assessments to validate the consistency of its dynamic changes and disease progression.

In summary, this study represents the first systematic analysis of the expression of the positive costimulatory molecules ICOS/ICOSL and negative costimulatory molecules PD-1/PD-L1 in membrane types and soluble forms in the peripheral blood of patients with NMOSD during an early stage prior to treatment. The results showed that the expression levels of mICOS/mICOSL and mPD-1/mPD-L1 were significantly higher in patients with NMOSD at an early stage compared with the ON, LETM, or HC groups. The sICOS level in patients with NMOSD was significantly lower than those in the other groups, whereas the sICOSL and sPD-1/sPD-L1 levels were significantly higher than those in the other groups. Consequently, excessive sICOSL levels were present in the blood, and positive signals were relatively dominant. These findings suggest that the expression levels of costimulatory molecules maybe have clinical value for the early differential diagnosis of NMOSD from LETM or ON, especially among AQP4-IgG-negative patients with NMOSD. Furthermore, from the perspective of the pathogenesis mechanism, these two pairs of costimulatory molecules participate in the pathological process of NMOSD, and their imbalanced expression might be an important pathological mechanism of NMOSD. How these two pathways exert different immunopathological effects across separate stages of the disease, how to choose the appropriate timing and method to intervene via key molecules, and whether the treatment of NMOSD during different stages can produce desirable effects remain important future research directions that are worthy of further investigation.

## Data Availability

All data generated or analyzed for this study are included in this published article (and its Supplementary Information files). The datasets generated during and/or analyzed during the current study are not publicly available because the research team is requesting additional funding; however, they are available from the corresponding authors on reasonable request.
