# Publisher Correction: Unbalanced Expression of ICOS and PD-1 in Patients with Neuromyelitis Optica Spectrum Disorder

**DOI:** 10.1038/s41598-020-64342-4

**Published:** 2020-05-08

**Authors:** Qun Xue, Xiaoping Li, Yanzheng Gu, Xiaozhu Wang, Mingyuan Wang, Jingluan Tian, Xiaoyu Duan, Hanqing Gao, Xiaopei Ji, Xiaoming Yan, Wanli Dong, Qi Fang, Xueguang Zhang

**Affiliations:** 1 Department of Neurology, First Affiliated Hospital of Soochow University, Suzhou, Jiangsu 215006 China; 2 Institute of Clinical Immunology, Jiangsu Key Laboratory of Clinical Immunology, First Affiliated Hospital of Soochow University, Suzhou, Jiangsu 215006 China; 3 Suzhou Clinical Medical Center of Neurology, Suzhou, Jiangsu 215004 China; 4 Suzhou Red Cross Central Blood Station, Suzhou, Jiangsu 215006 China

Correction to: *Scientific Reports* 10.1038/s41598-019-50479-4, published online 01 October 2019

This Article contains errors in the order of the Figures. Figures 1, 2, 3 and 4 were published as Figures 4, 1, 2 and 3 respectively. The correct Figures 1, 2, 3 and 4 appear below. The Figure legends are correct.

**Figure 1:**
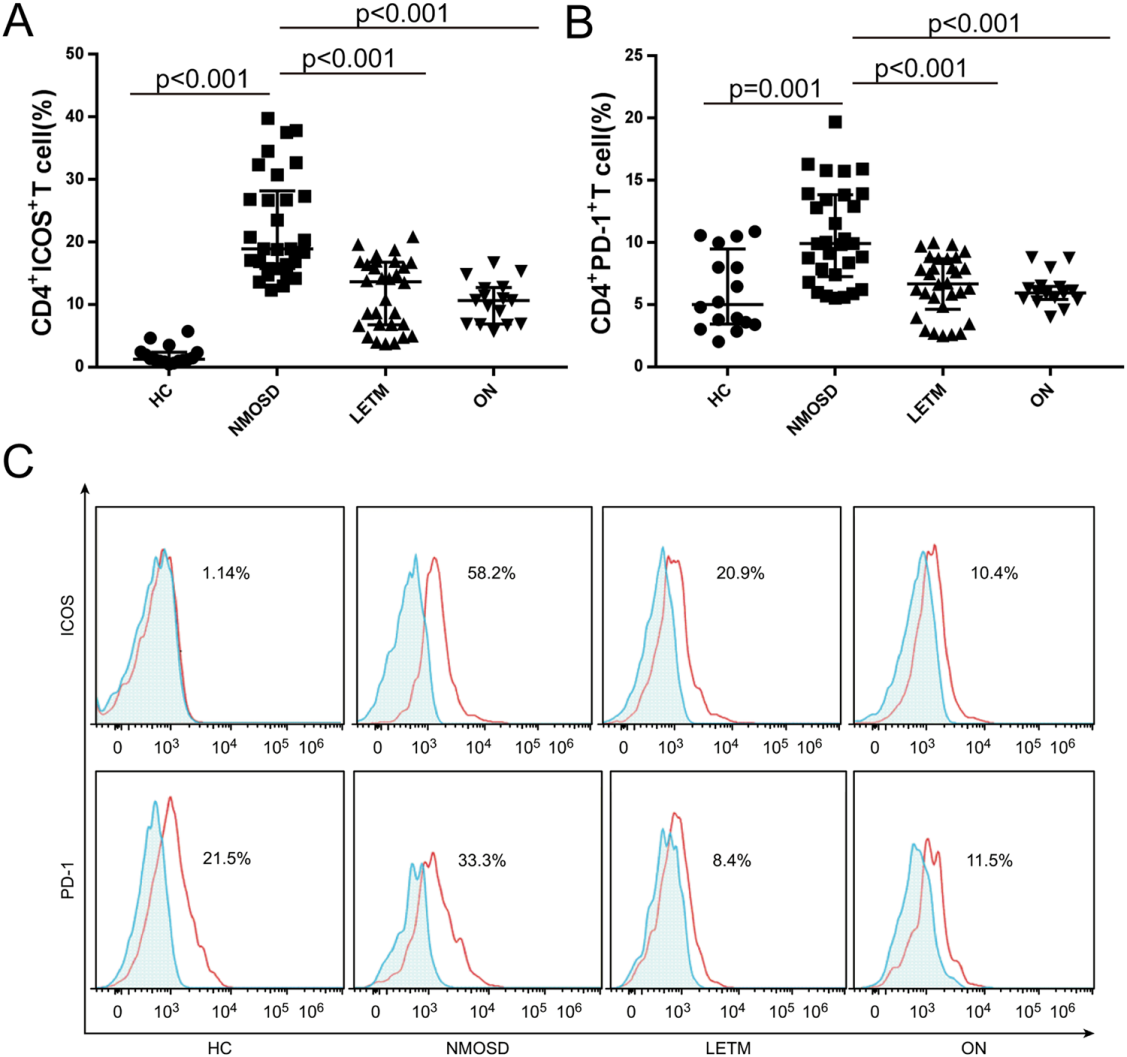
.

**Figure 2:**
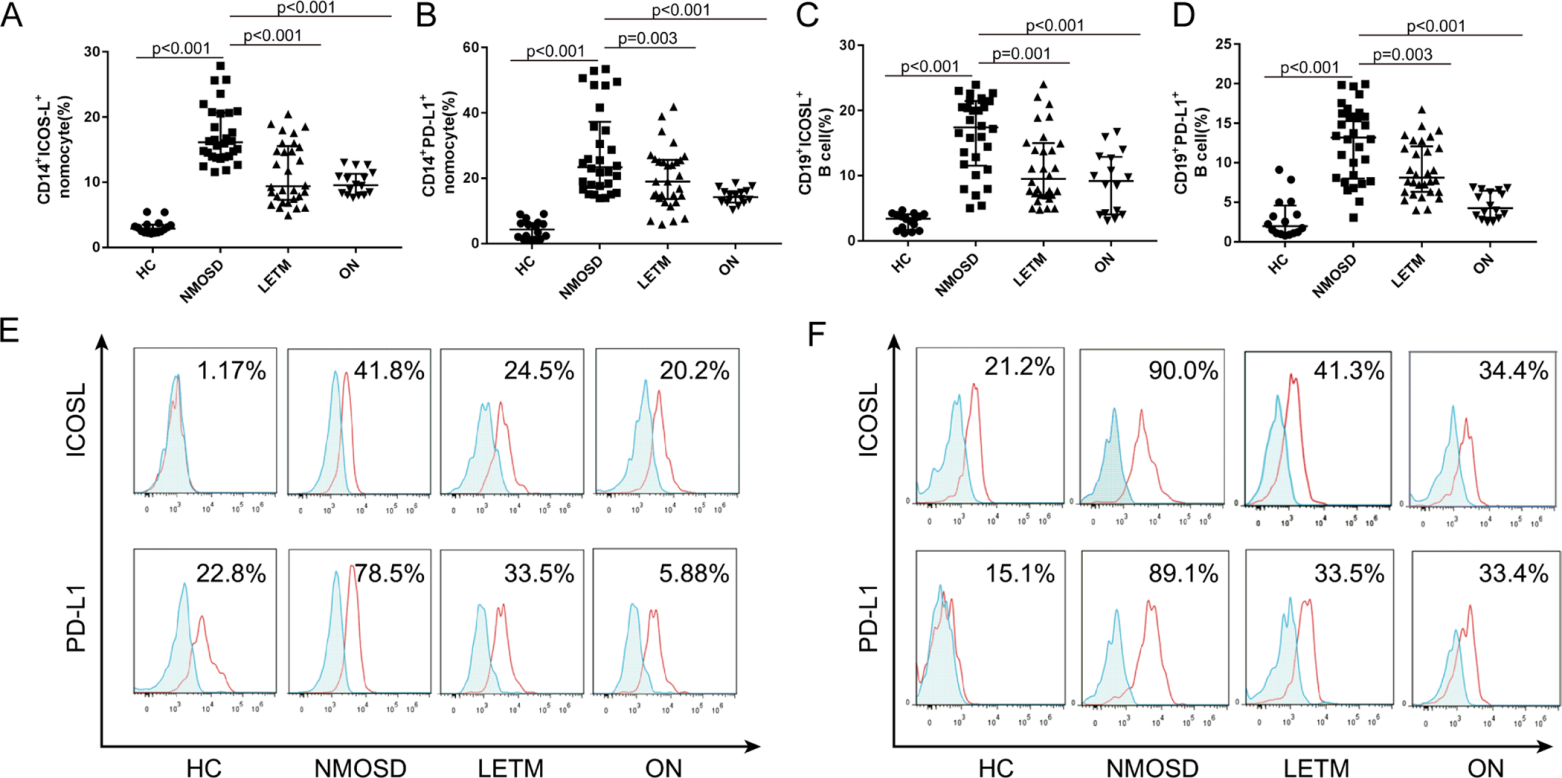
.

**Figure 3:**
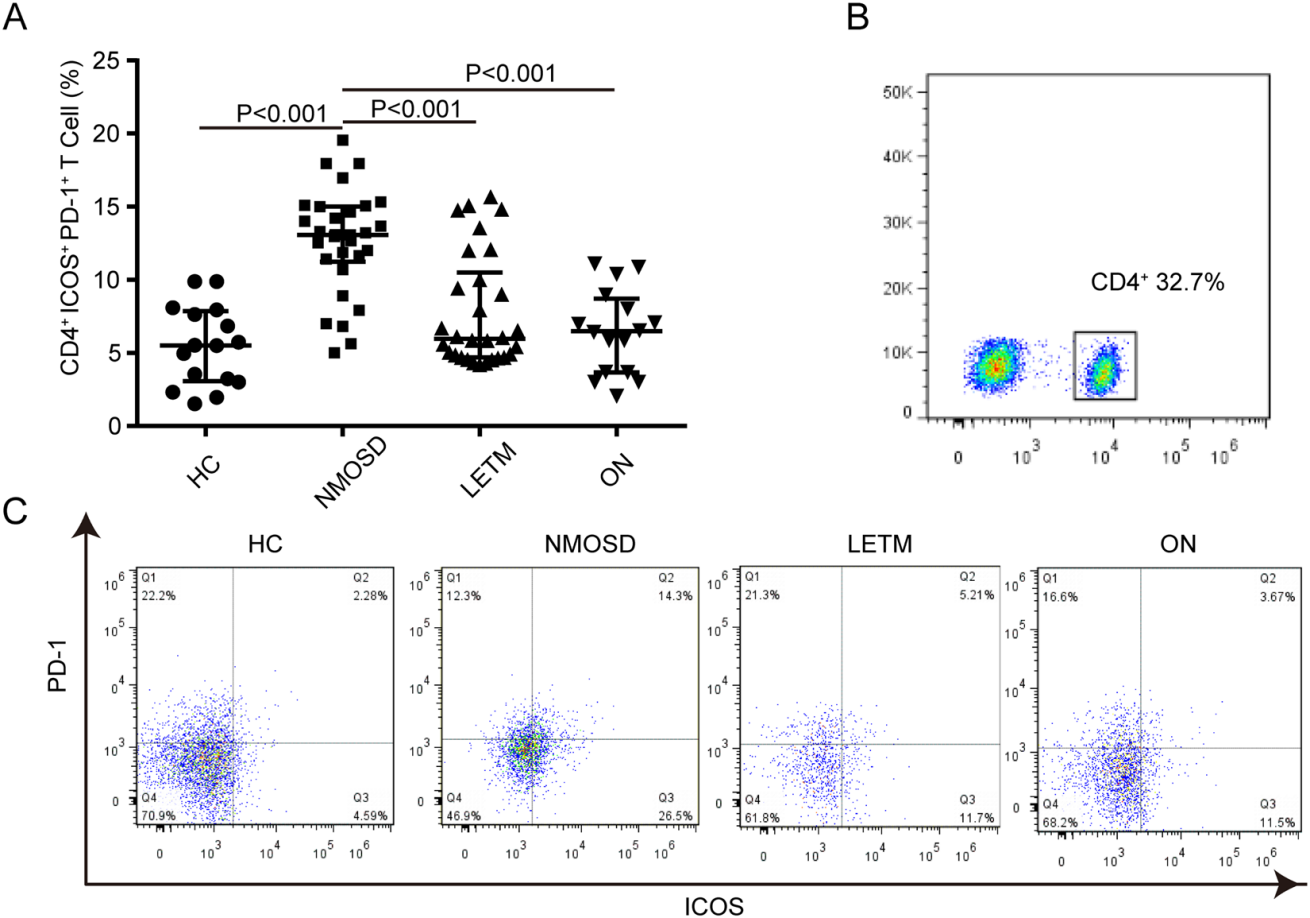
.

**Figure 4:**
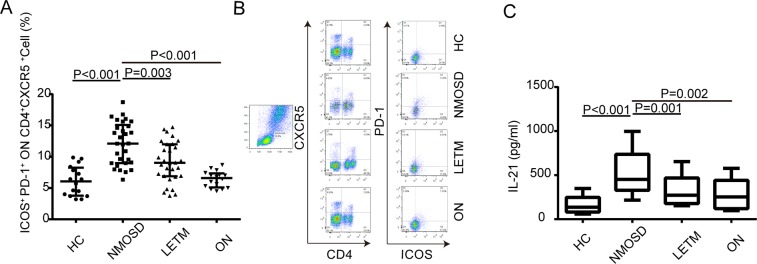
.

